# Online Pelvic Floor Group Education Program for Women With Persistent Genital Arousal Disorder/Genito-Pelvic Dysesthesia: Descriptive Feasibility Study

**DOI:** 10.2196/22450

**Published:** 2021-01-11

**Authors:** Robyn A Jackowich, Kayla M Mooney, Evelyn Hecht, Caroline F Pukall

**Affiliations:** 1 Department of Psychology Queen's University Kingston, ON Canada; 2 EMH Physical Therapy New York, NY United States

**Keywords:** persistent genital arousal disorder, genitopelvic dysesthesia, online program, pelvic floor, pilot

## Abstract

**Background:**

Persistent genital arousal disorder/genito-pelvic dysesthesia (PGAD/GPD) is a highly distressing yet poorly understood condition characterized by persistent genito-pelvic sensations, often described as “genital arousal,” which occur in the absence of sexual desire. PGAD/GPD is associated with significant impairment in psychosocial and daily functioning; however, there are currently no empirically validated treatment algorithms for PGAD/GPD. Pelvic floor physical therapy exercises have been found to be effective at reducing other forms of genito-pelvic discomfort, such as vulvodynia, and may also be beneficial to those experiencing PGAD/GPD. Many individuals with PGAD/GPD report difficulty finding a health care provider who is knowledgeable about PGAD/GPD; therefore, pelvic floor education and exercises in an online format may have the potential to reach more individuals in need.

**Objective:**

This study examined the feasibility of an online pelvic floor group education program; descriptively assessed outcomes related to distress, discomfort, catastrophizing, and mood; and obtained feedback from participants in order to inform the development of improved online group programs.

**Methods:**

Fourteen women with current symptoms of PGAD/GPD attended an online, 8-session pelvic floor group education program. Participants completed questionnaires of symptoms (ie, symptom distress, discomfort) and psychosocial well-being (ie, depression, anxiety, symptom catastrophizing) prior to the group sessions (Time 1), immediately after the final group session (Time 2), and 6 months following the final group session (Time 3). Participants also completed an anonymous feedback questionnaire immediately following the group program.

**Results:**

Overall, participants who attended a larger number of the group sessions (>5 sessions, n=7) appeared to report lower baseline (Time 1) symptoms and psychosocial impairment than those who attended fewer sessions (<5 sessions, n=7). A pattern of small improvements was seen following the group sessions on symptom and psychosocial outcomes. In the feedback questionnaire, breathing and relaxation exercises were described to be the most helpful home practice exercises, and participants rated sessions on (1) the relationship between emotions and PGAD/GPD symptoms and (2) relaxation exercises to be the most helpful. A number of barriers to participation in the group program were also identified, including comorbid health concerns and lack of personal time to complete the program/exercises.

**Conclusions:**

Online interventions provide an opportunity to reach international participants who may otherwise struggle to access a knowledgeable provider for their PGAD/GPD symptoms. Addressing barriers may help to increase participants’ abilities to engage in the program. Future programs may seek to integrate a greater focus on relaxation strategies and cognitive-affective strategies for managing PGAD/GPD symptoms.

## Introduction

Persistent genital arousal disorder/genito-pelvic dysesthesia (PGAD/GPD) is a highly distressing yet misunderstood condition characterized by distressing genito-pelvic sensations (ie, dysesthesias) often described as “genital arousal” [1]. Although many people assume that sensations of genital arousal are pleasant, wanted, and aligned with one’s internal sense of feeling “turned on”, PGAD/GPD represents a clear example of disagreement between the physical sensations of what would commonly occur in response to effective sexual stimulation and the subjective experience of those sensations. Part of the negative experience of these sensations is due to their extreme nature: the sensations are most often prolonged, persistent, and difficult—if not almost impossible—to stop [2]. In addition, they are commonly described as painful [1,3]. These sensations can occur in response to a variety of sexual and nonsexual triggers, or they may occur suddenly and unexpectedly [2]. Studies have indicated that levels of distress in response to these symptoms are predominantly moderate to high, defined as a mean of around 7 on a scale from 0 (no distress) to 10 (extremely high distress) [2,4].

Despite the recent emergence of clinical and research attention to this pattern of symptoms, the most commonly used diagnostic manuals do not yet include a formal diagnosis of PGAD/GPD, with the exception of the most recent version of the International Classification of Diseases (ICD-11 [5]). Additional efforts have been made, and the most recent classification system for sexual dysfunctions published by the International Society for the Study of Women’s Sexual Health (ISSWSH) includes criteria for PGAD/GPD [6]. The ISSWSH criteria are based on expert opinion and consist of the following: persistent or recurrent, unwanted or intrusive, distressing feelings of genital arousal, or being on the verge of orgasm, not associated with concurrent sexual interest, thoughts, or fantasies with a duration of 6 months or more. These feelings can be associated with: (a) limited resolution, no resolution, or aggravation of symptoms by sexual activity with or without aversive or compromised orgasm; (b) aggravation of symptoms by certain circumstances; (c) despair, emotional lability, catastrophizing, or suicidality; and (d) inconsistent evidence of genital arousal (eg, vaginal lubrication) during symptoms.

Although the prevalence of PGAD/GPD is unknown because of a lack of large-scale epidemiological studies, estimates from other sources exist. Based on their sample of women who presented at a sexual health clinic in the United Kingdom, Garvey and colleagues (2009) estimated that PGAD/GPD may affect approximately 1% of women [7]. More recently, three community samples from Canada, the United States, and Italy have found a similar prevalence rate, with 0.6% to 2.7% of women endorsing all of the characteristic features of PGAD/GPD at a moderate or higher frequency [8,9]. It is important to note that most of the clinical and research literature focuses on women with symptoms of PGAD/GPD; however, case studies describing similar symptoms in men have also emerged [10]. Given the significant representation of women in the current research literature, the research cited in this paper focuses on PGAD/GPD in women.

No empirically validated treatment algorithms for PGAD/GPD exist. Management commonly consists of pharmacological approaches, psychological interventions, and pelvic floor physical therapy [10], with some health care providers offering surgical interventions [11]. However, none of these treatment options have been formally tested or validated. A conservative approach to symptom management is often recommended, with the options being, in part, guided by the patients’ preferences and level of distress and the health care providers’ expertise and referral base [12]. Although there may be variations in the specific options and the timing of these options offered by various health care providers, many agree that the symptom-related distress must be specifically addressed in those with PGAD/GPD given the high frequency of self-reported suicidal ideation in this group [13]. However, access to treatment remains a barrier for many of those with PGAD/GPD due to the nature of the symptoms; many affected patients report shame and embarrassment surrounding the communication of their symptoms to others, including health care providers [14]. Even those who approach their health care providers may leave the situation feeling misunderstood and stigmatized because PGAD/GPD and its possible treatment options are not well known or understood [14]. Furthermore, the practical aspects of travelling to see a health care provider who is knowledgeable about PGAD/GPD may present major obstacles, ranging from financial constraints, to geographic barriers, to the challenges posed by travelling, which may significantly aggravate symptoms (eg, vibrations from car, prolonged sitting [2,14]).

In an effort to examine the feasibility of an accessible therapeutic option geared toward alleviating distress for those with PGAD/GPD, we piloted an online group program focusing on pelvic floor education and exercises and distress reduction for women with PGAD/GPD. Our focus on the pelvic floor in this group is based on a case study of a woman with PGAD/GPD who was successfully treated via pelvic floor rehabilitation [15] as well as on empirically based recommendations for other conditions characterized by genito-pelvic discomfort/dysesthesia (eg, vulvodynia [16]). Our aims were to (1) examine the feasibility of an online group program; (2) descriptively assess outcomes related to distress, discomfort, catastrophizing, and mood; and (3) obtain feedback from participants in order to inform the development of improved online group programs.

## Methods

### Participants

Participants were women who were experiencing symptoms of PGAD/GPD for a minimum of 3 months. The inclusion criteria for PGAD/GPD were based on its clinical descriptors [6,17]. PGAD/GPD includes experiencing feelings of persistent, involuntary genital arousal sensations that (1) are not fully relieved by one or more orgasms; (2) occur in the absence of subjective feelings of sexual arousal; (3) persist (ie, last longer than 30 minutes); (4) are experienced as intrusive and unwanted; and (5) are experienced as subjectively distressing.

In order to be eligible to participate, participants were also required to be 18 years of age or older, fluent in English, and not experiencing any other serious mental health concerns that would interfere with their ability to participate in the group sessions (examples included, but were not limited to, substance use disorder, borderline personality disorder, and psychosis). In order to determine the effectiveness of the program, participants were asked to not make any changes to their PGAD/GPD treatments or medications during the course of the 8-session weekly program.

**Figure 1:**
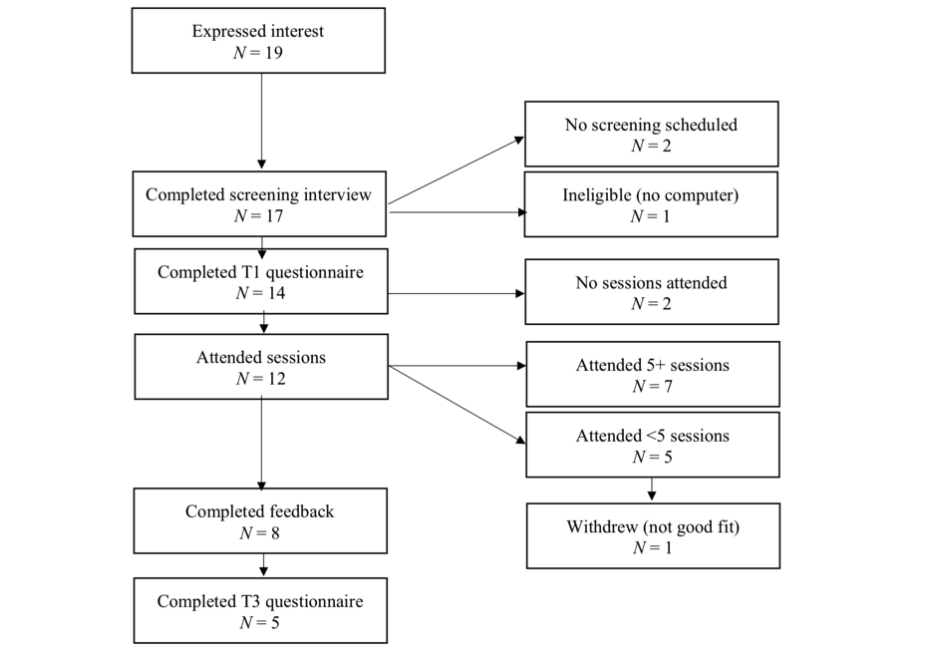
Flow of participation.

Participants were recruited online via social media advertisements (ie, Facebook, Twitter, blog posts) and postings on relevant websites and listservs to patients and health care professionals. Our laboratory also has a database of participants from past research studies who consented to be notified about additional research opportunities. Initially, 19 women contacted the study team to express interest in participating, and 17 underwent a phone screening with a member of the study team to confirm eligibility. See Figure 1 for a depiction of participant flow.

### Measures

**Figure 2:**
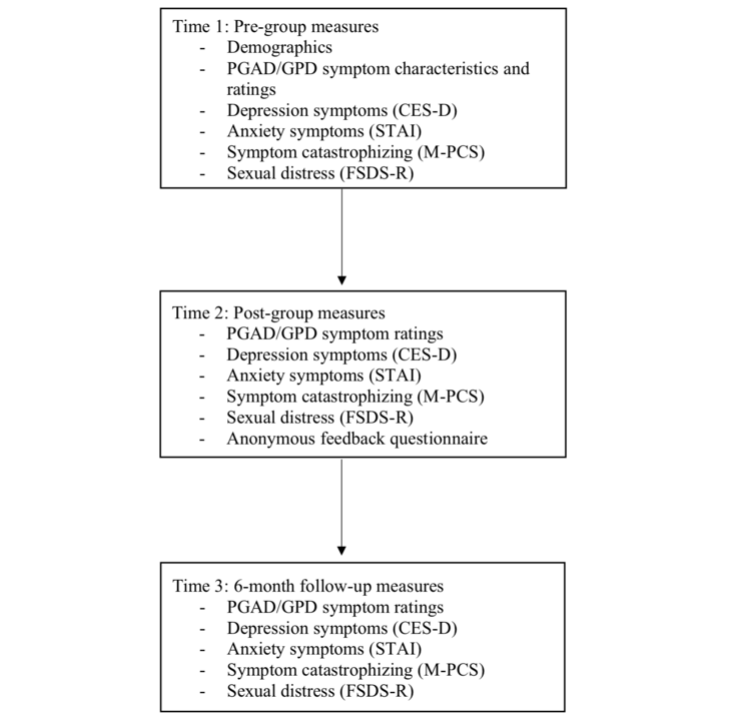
Self-report measures included at each time point of the study. PGAD/GPD: persistent genital arousal disorder/genito-pelvic dysesthesia.

Questionnaires were completed before the start of the group (Time 1), immediately after the final group session (Time 2), and 6 months following the final group session (Time 3). See Figure 2 for an outline of the measures included at the different time points of the program.

#### Demographic Information

Participants were asked to provide sociodemographic information, including age, ethnicity, education, occupation, relationship status, and sexual orientation.

#### Symptom Characteristics

Participants were asked to report on a number of PGAD/GPD symptom characteristics: the approximate date of their PGAD/GPD symptom onset, the proportion of time PGAD/GPD symptoms are present (*from 0% to 100%*), the distress associated with their PGAD/GPD symptoms (*0=none to 10=most distress ever*), and the discomfort associated with their PGAD/GPD symptoms (*0=none to 10=most discomfort ever*). Participants were also asked about other gynecological concerns that they experience and the number of health care providers that they had approached regarding their PGAD/GPD symptoms.

#### Depression Symptoms (Center for Epidemiologic Studies Depression Scale)

Symptoms of depression were assessed using the Center for Epidemiologic Studies Depression Scale (CES-D) [18]. The CES-D is a 20-item scale designed to assess the frequency of symptoms of depression over the past week. The measure is scored on a 4-point scale, with response options ranging from 0 (*Rarely, or none of the time; less than 1 day*) to 3 (*Most or all of the time; 5-7 days*). Higher scores on the CES-D represent more depressive symptoms.

#### Anxiety Symptoms (State-Trait Anxiety Inventory)

Symptoms of anxiety were assessed using the trait subscale of the State-Trait Anxiety Inventory (STAI) [19]. The STAI trait subscale is comprised of 20 statements. Participants were asked to rate how well each statement describes them on a 4-point scale. Response options range from 1 (*Almost never*) to 4 (*Almost always*). Higher scores on the STAI trait subscale indicate greater trait anxiety.

#### Symptom Catastrophizing (Modified Pain Catastrophizing Scale)

Symptom catastrophizing was measured using a modified version of the Pain Catastrophizing Scale (M-PCS) [20]. The PCS was originally designed to assess catastrophizing related to pain experiences but was modified for the purposes of this study by replacing the word “pain” with “vulvar sensations” throughout. The measure includes 13 statements that represent thoughts or feelings that may occur during PGAD/GPD experiences (eg, “I worry all the time about whether the vulvar sensations will end,” “I feel I can’t go on,” etc), and participants were asked to report on the frequency at which they experience these thoughts or feelings from 0 (*Not at all*) to 4 (*All the time*). Total M-PCS scores range from 0 to 52, with higher scores indicating higher levels of symptom catastrophizing. Scores of 30 and above on the original scale suggest clinical levels of catastrophizing [20].

#### Sexual Distress (Female Sexual Distress Scale – Revised)

The Female Sexual Distress Scale – Revised (FSDS-R) [21] was used to assess sexually-related personal distress. The measure contains a list of 13 feelings and problems that some people have regarding their sexuality (eg, distressed about your sex life, unhappy about your sexual relationship). Participants were asked to rate how often each problem had bothered them or caused them distress over the past 30 days, on a 5-point scale ranging from 0 (*Never*) to 4 (*Always*). Higher scores on the FSDS-R indicate greater levels of sexual distress.

#### Global Perception of Improvement

The two post-group questionnaires (Times 2 and 3) included a single-item question estimating percent overall improvement of PGAD/GPD symptoms (including emotional well-being, pain, sexual functioning, relationship, etc from 0% to 100%) as a result of attending the program.

#### Anonymous Feedback Questionnaire

A feedback questionnaire was administered to all participants who attended at least one of the group sessions following the final session of the program to gather information about the acceptability of the session format and content. Participants were asked which sessions they found the most and least helpful. Feedback was also solicited on the acceptability of the length and frequency of the sessions as well as on homework exercises, including the degree to which participants were able to complete the exercises (*from 0=not at all to 4=a high degree*), the most and least helpful exercises, and factors that would have facilitated homework completion. Participants also commented on the potential benefit of including partners in the group.

### Procedure

The study received approval from the Queen’s University (Kingston, ON) Health Sciences and Affiliated Hospitals Research Ethics Board. Following the telephone screening, eligible participants were directed to an online survey hosted by Qualtrics survey software to complete before the group began (Time 1). After reading a Letter of Information and providing their consent to participate, participants completed the survey, which took approximately 30 to 45 minutes.

The online group education program ran from January to March of 2018. The program included 8 weekly sessions, each lasting 120 minutes. The sessions were hosted on Zoom videoconferencing software. The Zoom software transmits only encrypted information, with multilayer security and end-to-end encryption (“Encryption for Meetings”, 2019). As such, Zoom is in accordance with the Health Insurance Portability and Accountability Act (HIPAA, 1996). Participants were provided with detailed instructions on how to use Zoom, and a team member (KMM) was present during all sessions to assist with any technical difficulties. Moreover, participants in the group were instructed to respect and maintain the confidentiality of other members of the group. The topics presented in each of the weekly education sessions are presented in Table 1.

**Table 1:** Topics covered in each of the online pelvic floor group education sessions.

Session number	Session topic
Session 1	Introduction to the science of pain (ie, processes that occur within the nervous system when one is in pain or discomfort [such as PGAD/GPD symptoms])
Session 2	Diaphragmatic breathing and its connection to the pelvic floor
Session 3	The benefits of movement and exercise on pelvic floor function
Session 4	Mindfulness
Session 5	Strategies for communicating one’s needs while experiencing discomfort, including tips for educating clinicians and sharing information with loved ones
Session 6	The role of nutrition in inflammation and experiences of discomfort
Session 7	Sleep hygiene and strength training (general body conditioning exercises to help a person with chronic symptoms become more functional in their activities of daily living)
Session 8	Emotion regulation and self-compassion

Each of the 8 sessions followed a set structure. First, participants were welcomed, and the educational topics for the session were introduced. Then, participants were guided to perform a breath technique to center/focus on the present. Following the breathing exercise, the educational topic of the session was presented by a registered physical therapist (EH), who then demonstrated the exercise introduced that session. Following the demonstration, the physical therapist then provided verbal guidance for participants to perform the exercise. Participants then engaged in a discussion about the topic, and the session ended with a question and answer period. There was no cost to participants for the group educational sessions, and no compensation was provided. When participants missed a session, they were provided with handouts summarizing the material presented in that session. Immediately following the completion of the online program, participants were sent a link to complete the online questionnaires for a second time (Time 2). Participants were also sent the same online questionnaires 6 months following completion of the program (Time 3).

## Results

### Data Considerations

The final sample contained 14 participants at the start of the group (Time 1), 6 participants at the end of the group (Time 2), and 5 participants at the 6-month follow-up (Time 3; see Figure 1).

#### Quantitative Results

Prior to conducting analyses, the data were examined for missing values and outliers where appropriate. No outliers were identified, defined as values more than 3 times the interquartile range [22]. No missing data were imputed for sociodemographic or symptom questions. On validated questionnaires with more than 10 items (CES-D, STAI, M-PCS, FSDS-R), if fewer than 15% of the items were missing for each individual, missing values were replaced with the individual’s mean response on that questionnaire. If more than 15% were missing, that individual’s questionnaire was excluded from the analyses. Quantitative results are presented as means and standard deviations. Analyses were conducted using IBM SPSS Version 26.

#### Feedback Questionnaire

Responses to open-ended questions in the feedback questionnaires are presented.

### Sample Demographics and Symptom Characteristics

Demographic and symptom characteristics for participants who completed the Time 1 questionnaires (n=14) are presented in Table 2. Participants were, on average, 43.71 years old (SD 17.65; range: 18 to 71). With respect to PGAD/GPD symptoms, participants reported a long average duration of symptoms (mean 7.43 years, SD 10.25), and a moderate to high level of associated distress and discomfort (Table 2).

**Table 2:** Sociodemographic information and symptom characteristics for participants who completed the pre-program questionnaires (Time 1; n=14).

Characteristic		Values
**Ethnicity, n (%)**		
	American	8 (57)
	Northern European (except British Isles)	3 (21)
	French	1 (7)
	Latin American	1 (7)
	American and Eastern European	1 (7)
**Occupational status, n (%)**		
	Full-time	2 (14)
	Part-time	5 (36)
	Unemployed	2 (14)
	Retired	1 (7)
	On disability	4 (29)
**Education, n (%)**		
	All/some high school	2 (14)
	All/some college/undergraduate degree	7 (50)
	All/some graduate school/professional training	5 (36)
**Relationship status, n (%)**		
	Single	1 (7)
	Dating	3 (21)
	Married/cohabitating	8 (57)
	Divorced	2 (14)
**Sexual orientation, n (%)**		
	Mixed-sex oriented	9 (64)
	Same-sex oriented	2 (14)
	Bisexual	2 (14)
	Asexual	1 (7)
**PGAD/GPD^a^ symptoms, mean (SD)**		
	Time since PGAD/GPD symptom onset, years	7.43 (10.25)
	Time PGAD/GPD present, %	63.31 (28.83)
	Distress (0=none, 10=most distress ever)	7.64 (2.27)
	Discomfort (0=none, 10=most discomfort ever)	6.43 (3.63)
	HCPs^b^ seen for PGAD/GPD, n	5.50 (5.40)
	Other gynecological concerns^c^, n	2.14 (1.99)

^a^PGAD/GPD: persistent genital arousal disorder/genito-pelvic dysesthesia.

^b^HCP: health care provider.

^c^Examples of other gynecological concerns include interstitial cystitis, pelvic inflammatory disease, endometriosis, and sexually transmitted infections.

### Who Attends the Majority of the Group Program?

Of those who completed the Time 1 questionnaire, 7 attended 5 or more of the sessions, and 7 attended less than 5 sessions. For most absences, no reason was provided; when reasons were provided, the most common ones were a scheduling conflict (9 absences) or being too sick/tired (5 absences). To better understand who attended the majority of the program sessions, health history, symptoms, and psychosocial well-being are presented for those who attended 5 or more versus less than 5 sessions (Table 3). Visual inspection of the data suggested that those who attended 5 or more sessions were younger, reported more gynecological comorbidities, and reported less severe PGAD/GPD symptoms (lower associated discomfort, lower associated distress, and symptoms were present for shorter amount of time). They also reported lower baseline depressive and anxiety symptoms and lower symptom catastrophizing; however, they reported greater sexual distress. Overall, it appears that those with less severe PGAD/GPD symptoms and associated psychosocial concerns attended the majority of the program sessions. Graphs representing all individual responses for each individual participant are presented in Multimedia Appendix 1.

**Table 3:** Average scores (and SD) of participants who attended 5 or more of the 8 online educational group sessions (n=7) and those who attended less than 5 sessions (n=7).

Characteristic	Attended 5+ sessions, mean (SD)	Attended <5 sessions, mean (SD)
Age, years	41.7 (16.3)	45.7 (20.0)
Other gynecological concerns, n	2.4 (1.5)	1.9 (2.5)
Time PGAD/GPD^a^ present, %	59.8 (33.7)	66.3 (26.4)
Distress score	7.6 (2.5)	7.7 (2.2)
Discomfort score	5.4 (3.9)	7.4 (3.4)
CES-D^b^	26.9 (11.9)	32.3 (12.6)
STAI^c^	54.3 (14.1)	58.7 (15.3)
M-PCS^d^	32.5 (13.7)	34.7 (13.3)
FSDS-R^e^	33.4 (14.0)	29.3 (8.3)

^a^PGAD/GPD: persistent genital arousal disorder/genito-pelvic dysesthesia.

^b^CES-D: Center for Epidemiologic Studies Depression Scale.

^c^STAI: State-Trait Anxiety Inventory.

^d^M-PCS: Modified Pain Catastrophizing Scale.

^e^FSDS-R: Female Sexual Distress Scale – Revised.

### Symptoms and Psychosocial Well-being Before and After the Program

At Time 2, participants rated their overall perceived improvement to be 13.5% (SD 20.29; range: 0% to 50%; n=6). At Time 3, participants rated their overall perceived improvement to be slightly higher (mean 15.0%, SD 11.18; range: 0% to 30%; n=5). Descriptive information about symptom characteristics, psychosocial adjustment, and sexual well-being at all 3 time points is presented in Table 4. An overall pattern emerged, such that PGAD/GPD symptoms and psychosocial well-being improved across time, with the exception of discomfort associated with PGAD/GPD symptoms, which increased at Time 2 but decreased at Time 3. These results are consistent with the small improvements reported on the global improvement measure. Depression symptoms, anxiety symptoms, and sexual distress decreased following the group; however, the average scores remained within the range indicating clinically significant levels. The average score of symptom catastrophizing (M-PCS) fell in the range indicating clinically significant catastrophizing at Time 1 but decreased at Times 2 and 3 to a score that no longer fell in the range indicating clinical significance. Individual responses on each of the outcome variables, plotted across the 3 study time points, are presented in Multimedia Appendix 2.

**Table 4:** Average scores prior to attending the online educational group program (Time 1), at the end of the 8-week group program (Time 2), and 6 months following the program (Time 3).

Characteristic	Time 1, mean (SD) n	Time 2, mean (SD) n	Time 3, mean (SD) n
Time PGAD/GPD^a^ present, %	63.31 (28.83) 13	54.83 (34.96) 6	51.25 (33.26) 4
Distress score	7.64 (2.27) 14	7.67 (3.14) 6	6.80 (2.39) 5
Discomfort score	6.43 (3.63) 14	7.33 (2.58) 6	5.40 (2.97) 5
CES-D^b^	29.57* (12.12) 14	28.67* (12.04) 6	23.0* (8.28) 5
STAI^c^	56.5* (14.34) 14	52.17* (13.41) 6	46.80* (12.85) 5
M-PCS^d^	34.21* (12.60) 14	29.67 (13.71) 6	25.40 (11.61) 5
FSDS-R^e^	31.36* (11.27) 14	30.33* (17.34) 6	19.00* (15.75) 5

^a^PGAD/GPD: persistent genital arousal disorder/genito-pelvic dysesthesia.

^b^CES-D: Center for Epidemiologic Studies Depression Scale.

^c^STAI: State-Trait Anxiety Inventory.

^d^M-PCS: Modified Pain Catastrophizing Scale.

^e^FSDS-R: Female Sexual Distress Scale – Revised.

^*^Scores that fall above established cutoffs, suggesting clinically significant symptoms.

### Feedback Questionnaire: What Did Participants Think About the Group Program?

At Time 2, 8 participants completed an anonymous feedback questionnaire about their experience attending the program. Overall, breathing and relaxation exercises were described to be the most helpful home practice exercises by the majority of participants (n=7). On average, participants reported a moderate ability to complete the home practice exercises (mean 2.0, SD 0.9). Participants reported that the most helpful topics were sessions that (1) discussed the relationship between emotions, discomfort, and PGAD/GPD symptoms (n=3) and (2) included relaxation exercises (ie, breathing, visualization; n=2). The sessions that were rated as least helpful were those on (1) nutrition (n=2) and (2) sleep and strength training (n=1). Ratings of mindfulness and the science of pain/discomfort received a mixed response (1 positive and 1 negative rating for each). All participants reported that they were happy with the number and length of the sessions, although one additionally specified that they would prefer biweekly sessions. Only 1 participant indicated that the inclusion of partners in the group would be helpful (no: n=5; not sure: n=2).

Open-ended responses on the feedback questionnaire are presented in Textbox 1. When asked what was helpful about the sessions, themes emerged of *normalization* (eg, “not as alone as I feel”), *support to complete ongoing interventions* for the PGAD/GPD symptoms (“reminders of some of what I was already doing,” “having a focus each week”), and *hopefulness* (“made me once again think about what I can do to handle this”). When asked what would help participants to complete the home practice exercises regularly, 2 participants identified barriers to completing the exercises: *timing of the sessions* and *health concerns*. Two participants offered concrete changes to the structure of home practice exercises: a brief note summarizing the home practice exercises at the end of each session (n=1) and more personal interaction during the program (n=1).

**Open-ended responses from participants (n=8) who attended the 8-session group educational program.**

**Question 1: In what ways did you find the sessions helpful? What did you find most useful?**

- Knowing about how the brain works
- Reminder that emotional suffering, anxiety etc. can worsen physical conditions. Support that I am not as ‘alone’ as I feel trying to ENDURE this horrid condition!
- I appreciate having a focus each week and the science and research presented behind the methods.
- I learned something new in every session. The handouts after the sessions were over. I found that I was able to underline certain points and it was easier to look back over. I also liked the videos as I am a very visual learner.
- Made me once again think about what I can do to handle this while I still hope to get help from the doctors (that they will find a reason for PGAD/GPD).
- I knew most of the information already...
- Being able to see how others responded and what input they had was very valuable to me.
- Reminders of some of what I was already doing: meditations, yoga, physical exercise, correct nutrition.

**Question 2: What would have made it easier for you to complete the home exercises?**

- More personal interaction.
- More personal time. The sessions came at a very busy time in my life […] I didn't have the capacity to slow down.
- I think timing had a lot to do with issues for me. Having had two hospitalizations within a month […] has left me severely restricted at this time.
- A separate note with just the exercise after each session.
- Better health.

## Discussion

### Principal Findings

To our knowledge, this study is the first to examine the feasibility of an online pelvic floor group education program for PGAD/GPD. PGAD/GPD is associated with significant negative psychosocial impact [13], and there is great need for empirically based treatment interventions that address both PGAD/GPD symptoms and their consequences [10]. Given the limited information on and treatments available for PGAD/GPD [10,14], online interventions have the opportunity to reach many individuals who may not otherwise have access to treatment for PGAD/GPD. There is growing evidence that web-based health interventions can be effective in promoting knowledge and behavioral change in the management of other chronic illnesses [23].

Descriptive results regarding participants who attended the majority of the group sessions suggested that they were younger and reported less severe PGAD/GPD symptoms and associated psychosocial concerns (eg, symptom catastrophizing, depression, and anxiety) than those who attended fewer sessions. In addition, those who attended the majority of the sessions and completed the Time 2 and 3 outcome measures reported small improvements in PGAD/GPD symptoms (proportion of time present, distress, and discomfort) and psychosocial well-being (depression symptoms, anxiety symptoms, catastrophizing of PGAD/GPD symptoms, and sexual distress). These results indicate that regular participation in an online group program may be beneficial in terms of outcome. However, these findings also suggest that, even when intervention is presented via an online format, participants with more severe PGAD/GPD symptoms and associated psychosocial consequences may still face barriers to engaging in such an intervention.

Indeed, previous studies have found that PGAD/GPD symptoms interfere with daily activities, such as sitting for prolonged periods of time and the ability to concentrate [2]. This interference may have prevented those with more severe symptoms from more fully attending the intervention-based sessions, even remotely. Future intervention programs for PGAD/GPD should consider modifications and accommodations that would help to address barriers to participation. For example, the integration of asynchronous content to allow participants more flexibility in the timing of some of the more educational aspects of the program, in combination with shorter synchronous sessions, with frequent rest breaks for the more experiential and interactive components of the program, may be helpful. More significant psychosocial correlates of PGAD/GPD symptoms (ie, greater depression symptoms, anxiety symptoms, and symptom catastrophizing) could interfere with participation by reducing motivation to attend sessions and complete home exercises or by increasing avoidance of discussions or exercises that may be perceived to increase symptoms. Individuals experiencing significant symptoms of depression, anxiety, or both may benefit from strategies to reduce depression/anxiety prior to, or concurrently with, pelvic health education.

We also collected feedback on the most and least helpful aspects of the program, with a view to use the knowledge gained to aid in the redevelopment of an online group intervention for those with PGAD/GPD. Based on the feedback, sessions that focused on stress management and the role of cognitions and emotions in the management of genito-pelvic discomfort and unwanted arousal seemed most helpful and should be included in future programs. Treatment approaches that focus on these aspects have been found to be effective for reducing pain and associated psychosocial difficulties in women with other forms of genito-pelvic discomfort (eg, vulvodynia [24-26]). Integration of these components early in the program may also help to address some of the barriers (ie, reducing distress and improving psychosocial well-being) to attending the program. Overall, in the feedback questionnaires, participants highlighted the value of normalization, hopefulness, and motivation/support to continue seeking treatments. The group format may be particularly valuable for individuals experiencing a condition, such as PGAD/GPD, that is surrounded by high levels of shame and hopelessness [3,13], and is often unknown within the health care community [14].

### Limitations

The results of this study must be considered in the context of several limitations. This study was descriptive in nature and relied on a small sample size. Future studies may seek to conduct a similar program in a larger sample, while addressing the barriers identified in this study. In addition, participants did not undergo a clinical exam to confirm their diagnosis of PGAD/GPD. However, an extensive phone screening interview reviewing the diagnostic criteria for PGAD/GPD was conducted, and previous research has found high agreement between self-reported symptoms and clinical diagnosis for samples of women with other forms of genito-pelvic discomfort (ie, vulvodynia [27,28]). Finally, there is no information about how PGAD/GPD symptoms change over time without intervention to use as a comparison to these results. While this sample reports a long duration of symptoms (7.43 years on average), the chronicity of PGAD/GPD is unknown. Research on other forms of genito-pelvic discomfort (ie, vulvodynia) has found that chronicity is heterogenous [29-31]. These studies also indicate that individuals with a longer duration of symptoms are more likely to report greater pain intensity, anxiety, comorbid chronic pain conditions, and a primary symptom onset [29-31]. More information about the chronicity of PGAD/GPD symptoms will aid in interpreting future treatment outcome research. Finally, the sample was limited to individuals with access to the Internet and a computer. Online interventions may have the ability to reach individuals who cannot travel for in-person interventions; however, a limitation is access to, knowledge of, and comfort with telehealth technology (such as videoconferencing software).

### Conclusions

Online interventions provide an opportunity to reach international participants who may otherwise struggle to access a knowledgeable provider for their PGAD/GPD symptoms. The group format may also help to encourage hopefulness and normalize the experience of symptoms that are often surrounded by feelings of shame. This study is the first online intervention study for PGAD/GPD, and it describes the feasibility of an 8-session pelvic floor education program. Overall, participants were satisfied with the length and frequency of the sessions. Deep breathing and relaxation exercises were reported to be beneficial by almost all participants. A number of barriers to participating in the program were identified (greater symptom and psychosocial impairment, timing of the sessions, concurrent health concerns) that could be addressed to help improve the efficacy of future interventions and increase the ability of participants to fully engage in the program. Future programs for PGAD/GPD may increase their focus on stress management strategies and working with thoughts and emotions related to PGAD/GPD symptoms. Finally, the results reinforce that PGAD/GPD is a highly distressing condition associated with significant burden. More research is needed to identify treatments and interventions to support individuals with PGAD/GPD.

## Appendix

Multimedia Appendix 1 Individual pre- and post-program self-report measures.

Multimedia Appendix 2 Individual changes in outcome variables across the three study time points.

## Abbreviations

CES-D: Center for Epidemiologic Studies Depression Scale
FSDS-R: Female Sexual Distress Scale – Revised
ISSWSH: International Society for the Study of Women’s Sexual Health
M-PCS: Modified Pain Catastrophizing Scale
PGAD/GPD: persistent genital arousal disorder/genito-pelvic dysesthesia
STAI: State-Trait Anxiety Inventory
