# Supplementary material for: Online Pelvic Floor Group Education Program for Women With Persistent Genital Arousal Disorder/Genito-Pelvic Dysesthesia: Descriptive Feasibility Study
Source: JMIR Form Res. 2021 Jan 11;5(1):e22450. doi: 10.2196/22450 (PMC7834936; doi:10.2196/22450)
Supplement: Multimedia Appendix 1 [file formative_v5i1e22450_app1.docx]

APPENDIX 1

Individual scores for each participant (n=14) on all pre-program measures (Time 1). The purple box plots represent individuals who attended 5 or fewer sessions of the group program, and the yellow box plots represent individuals who attended 5 or more sessions of the group program. The Y-axis represents scores on each of the variables examined (age, distress, discomfort, etc.). Each black off-center point represents an individual response. Larger, centered black points indicate when a score is an outlier. Note: PGAD/GPD = Persistent Genital Arousal Disorder/Genito-Pelvic Dysesthesia.


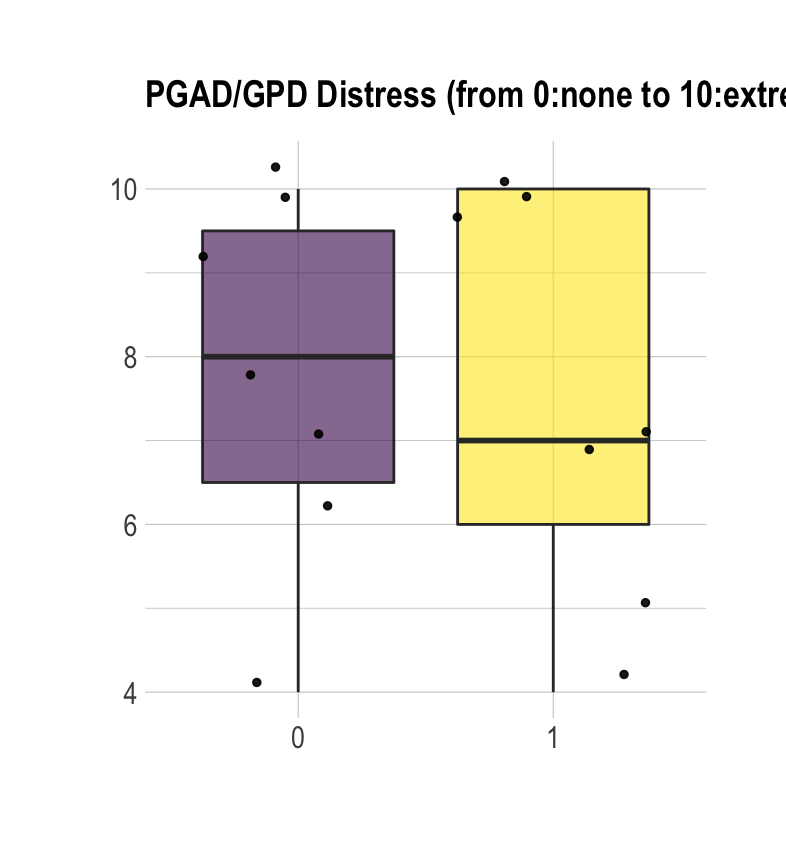

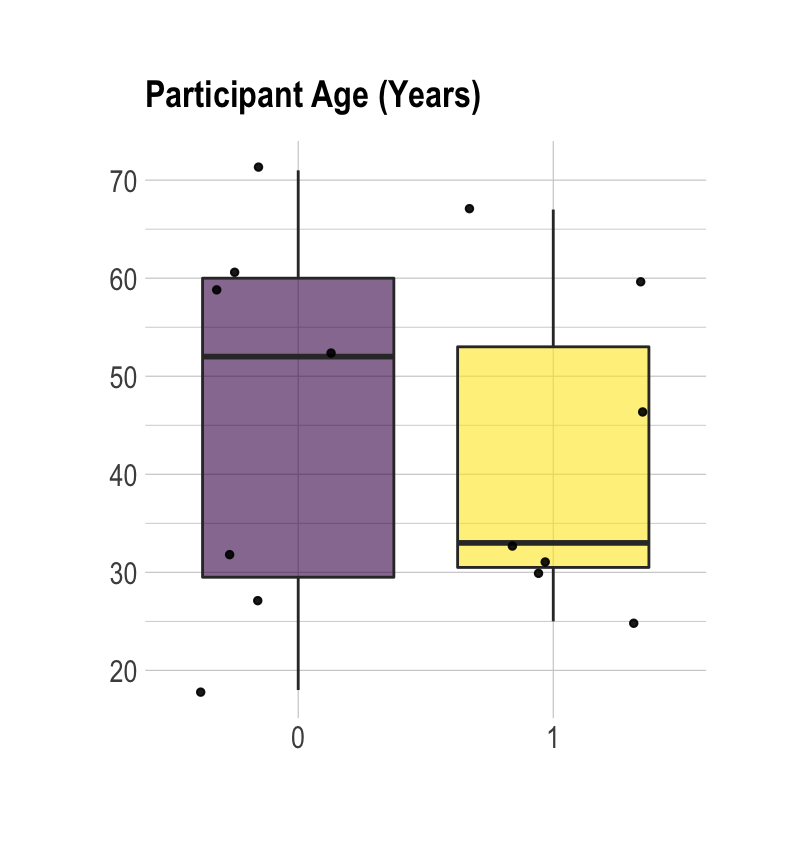


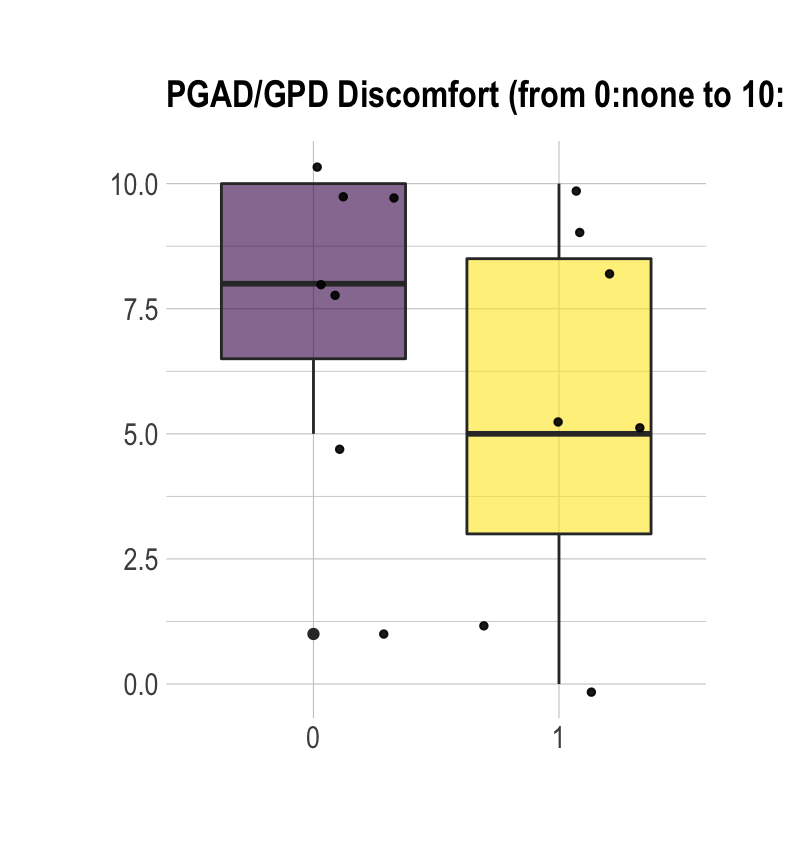


<5 Sessions >5 Sessions

Attended Attended

<5 Sessions >5 Sessions

Attended Attended

<5 Sessions >5 Sessions

Attended Attended

PGAD/GPD Distress

From 0 (none) to 10 (extreme)

PGAD/GPD Discomfort

From 0 (none) to 10 (extreme)

Participant Age (Years)


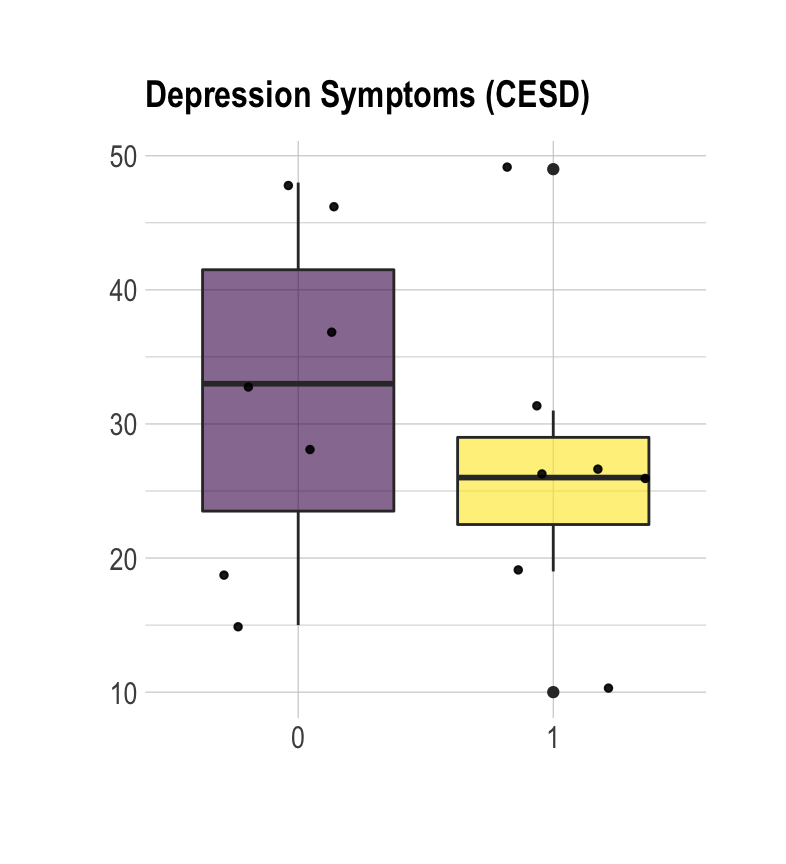

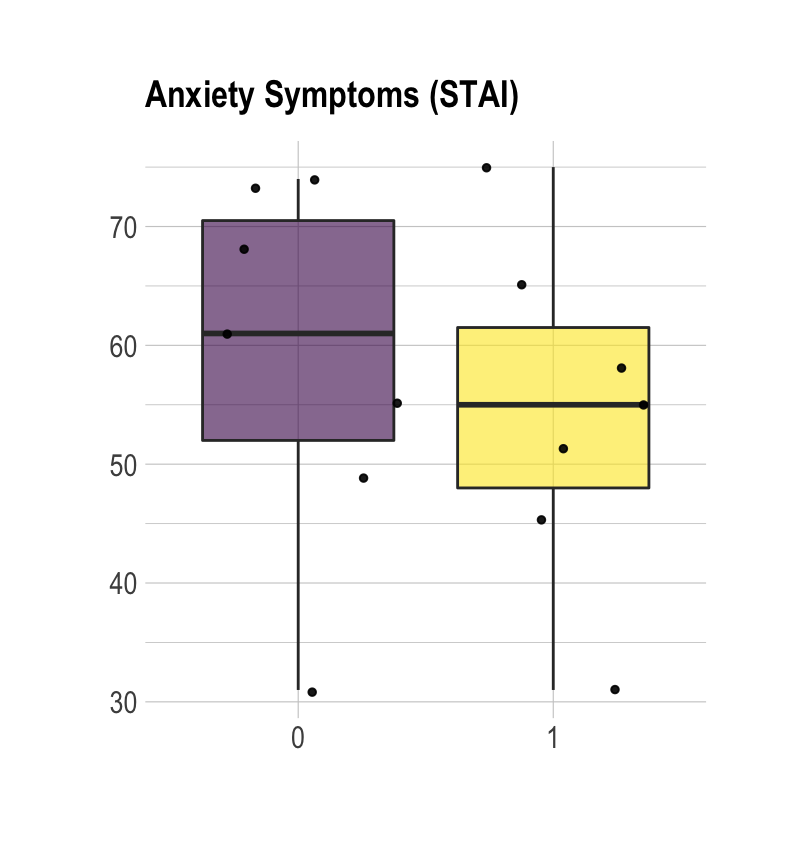

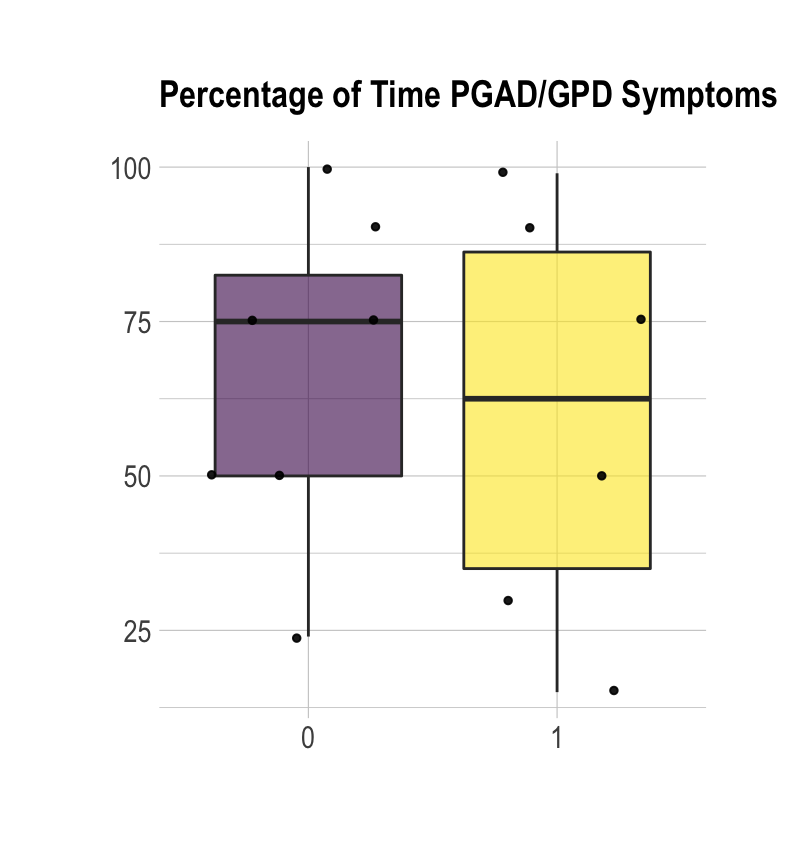

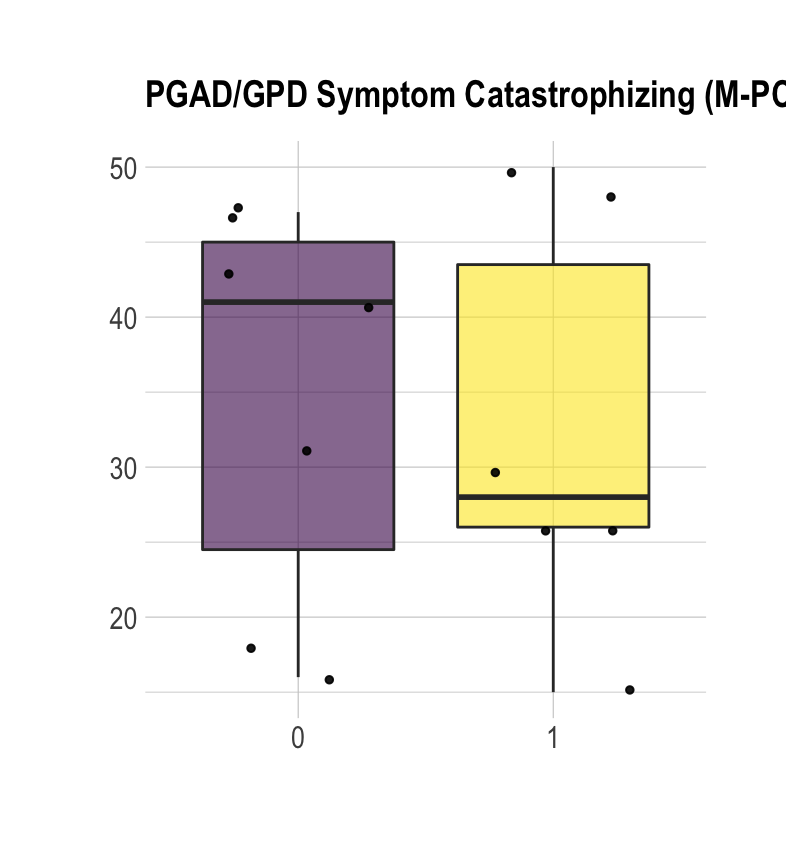

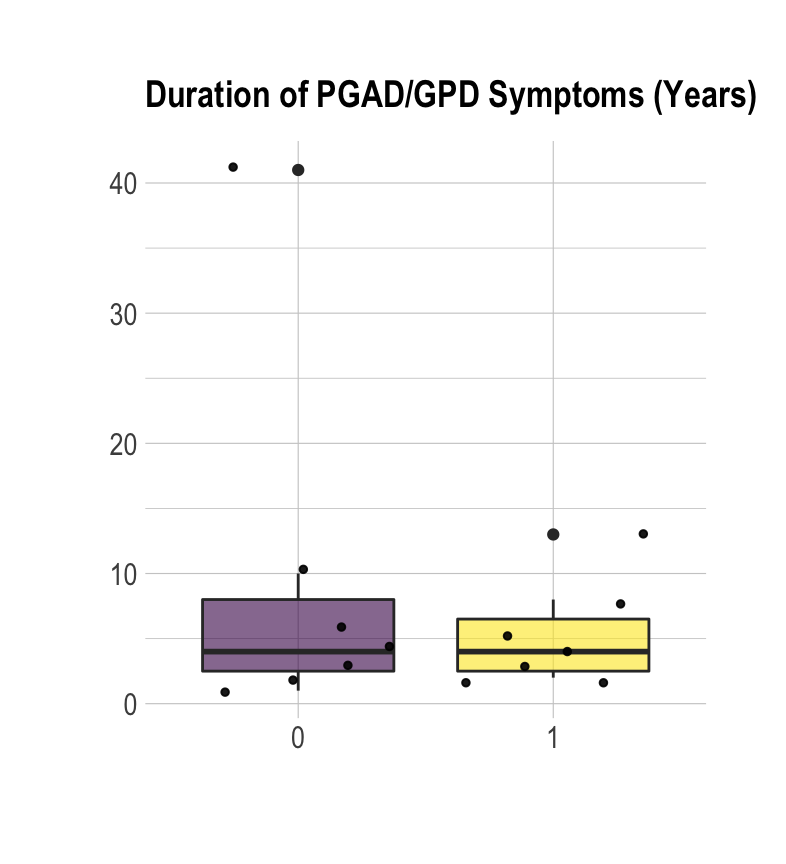


<5 Sessions >5 Sessions

Attended Attended

<5 Sessions >5 Sessions

Attended Attended

Anxiety Symptoms (STAI)

Depression Symptoms (CESD)

<5 Sessions >5 Sessions

Attended Attended

<5 Sessions >5 Sessions

Attended Attended

Percentage of Time PGAD/GPD Symptoms Are Present

Duration of PGAD/GPD Symptom (Years)

PGAD/GPD Symptom Catastrophizing (M-PCS)

<5 Sessions >5 Sessions

Attended Attended


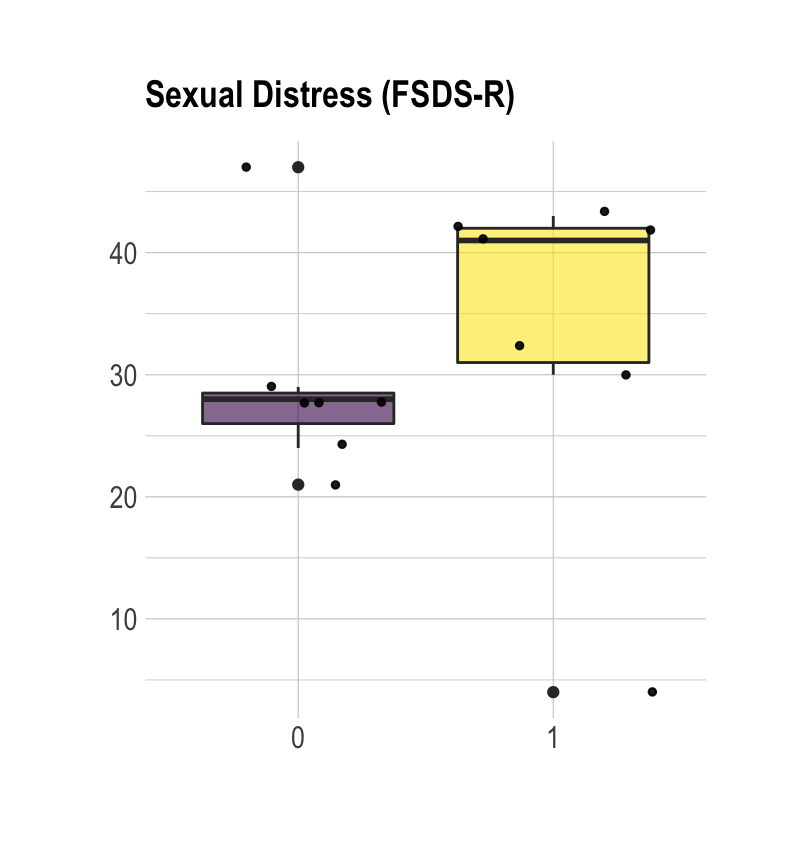


Sexual Distress (FSDS-R)

<5 Sessions >5 Sessions

Attended Attended

**APPENDIX 1.** Individual scores for each participant (*n* =14) on all pre-program measures (Time 1). The purple box plots represent individuals who attended 5 or fewer sessions of the group program, and the yellow box plots represent individuals who attended 5 or more sessions of the group program. The Y axis represents scores on each of the variables examined (age, distress, discomfort, etc.). Each black off-center point represents an individual response. Larger, centered black points indicate when a score is an outlier.

Note: PGAD/GPD = Persistent Genital Arousal Disorder/Genito-Pelvic Dysesthesia.
