# Supplementary material for: Online Pelvic Floor Group Education Program for Women With Persistent Genital Arousal Disorder/Genito-Pelvic Dysesthesia: Descriptive Feasibility Study
Source: JMIR Form Res. 2021 Jan 11;5(1):e22450. doi: 10.2196/22450 (PMC7834936; doi:10.2196/22450)
Supplement: Multimedia Appendix 2 [file formative_v5i1e22450_app2.docx]

APPENDIX 2

Each graph depicts individual changes (represented by different colors) in outcome variable (sexual distress, depression symptoms, anxiety symptoms, symptom catastrophizing, distress, discomfort, and % time persistent genital arousal disorder/genito-pelvic dysesthesia [PGAD/GPD] symptoms present) across the three study time points: time 1 (pre-program), time 2 (immediately after the final group session) and time 3 (6-months following the final group session). The majority of respondents saw a reduction in sexual distress (n=5/7), a reduction in anxiety symptoms (n=6/7); and a reduction in symptom catastrophizing (n=5/6). Results on depressive symptoms, symptom distress and discomfort were mixed (i.e., some participants reporting decreases, some increases, and some reporting no change. The percentage of time that PGAD/GPD symptoms are present showed a lot of movement across each of the time points – and speaks to the need for future research examining how PGAD/GPD symptoms change over time.

**APPNEDIX 2.** Each graph depicts individual changes (represented by different colors) in outcome variable (sexual distress, depression symptoms, anxiety symptoms, symptom catastrophizing, distress, discomfort, and % time persistent genital arousal disorder/genito-pelvic dysesthesia [PGAD/GPD] symptoms present) across the three study timepoints: time 1 (pre-program), time 2 (immediately after the final group session) and time 3 (6-months following the final group session). The majority of respondents saw a reduction in sexual distress (*n* = 5/7), a reduction in anxiety symptoms (*n* = 6/7); and a reduction in symptom catastrophizing (*n* = 5/6). Results on depressive symptoms, symptom distress and discomfort were mixed (i.e., some participants reporting decreases, some increases, and some reporting no change. The percentage of time that PGAD/GPD symptoms are present showed a lot of movement across each of the time points – and speaks to the need for future research examining how PGAD/GPD symptoms change over time.
